# Diagnostic performance of the Japanese Narrow-band imaging expert team classification system using dual focus magnification in real-time Vietnamese setting

**DOI:** 10.1097/MD.0000000000038752

**Published:** 2024-07-05

**Authors:** Nhan Quang Le, Tien Manh Huynh, Diem Thi Ngoc Vo, Huy Minh Le, Truc Thanh Le Tran, Vy Thao Ly Tran, Luan Minh Dang, Nghia Quang Le

**Affiliations:** a Department of GI Endoscopy, University Medical Center Ho Chi Minh City, Ho Chi Minh City, Viet Nam; b Department of Internal Medicine, University of Medicine and Pharmacy at Ho Chi Minh City, Ho Chi Minh City, Viet Nam; c Department of Histology – Embryology and Pathology, University of Medicine and Pharmacy at Ho Chi Minh City, Ho Chi Minh City, Viet Nam; d Department of GI Surgery, University of Medicine and Pharmacy at Ho Chi Minh City, Ho Chi Minh City, Viet Nam.

**Keywords:** colorectal polyp, dual focus, Japan narrow band imaging expert team, narrow-band imaging, Vietnam

## Abstract

The JNET classification, combined with magnified narrowband imaging (NBI), is essential for predicting the histology of colorectal polyps and guiding personalized treatment strategies. Despite its recognized utility, the diagnostic efficacy of JNET classification using NBI with dual focus (DF) magnification requires exploration in the Vietnamese context. This study aimed to investigate the diagnostic performance of the JNET classification with the NBI-DF mode in predicting the histology of colorectal polyps in Vietnam. A cross-sectional study was conducted at the University Medical Center in Ho Chi Minh City, Vietnam. During real-time endoscopy, endoscopists evaluated the lesion characteristics and recorded optical diagnoses using the dual focus mode magnification according to the JNET classification. En bloc lesion resection (endoscopic or surgical) provided the final pathology, serving as the reference standard for optical diagnoses. **A total of 739 patients with 1353 lesions were recruited between October 2021 and March 2023.** The overall concordance with the JNET classification was 86.9%. Specificities and positive predictive values for JNET types were: type 1 (95.7%, 88.3%); type 2A (81.4%, 90%); type 2B (96.6%, 54.7%); and type 3 (99.9%, 93.3%). The sensitivity and negative predictive value for differentiating neoplastic from non-neoplastic lesions were 97.8% and 88.3%, respectively. However, the sensitivity for distinguishing malignant from benign neoplasia was lower at 64.1%, despite a specificity of 95.9%. Notably, the specificity and positive predictive value for identifying deep submucosal cancer were high at 99.8% and 93.3%. In Vietnam, applying the JNET classification with NBI-DF demonstrates significant value in predicting the histology of colorectal polyps. This classification guides treatment decisions and prevents unnecessary surgeries.

## 1. Introduction

Colorectal polyps are closely associated with colorectal cancer, emphasizing the importance of early detection and identification of high-risk polyps to prevent and reduce mortality rates.^[1]^ The more inaccurate the diagnosis, the more unnecessary polypectomies and the higher medical costs the patients can encounter.^[2]^ Therefore, efforts are underway to improve the precise and real-time endoscopic diagnosis of colorectal neoplasms. Narrow-band imaging (NBI) endoscopy uses specific light wavelengths to assess the microstructure and surface microvasculature of the lesions. This capability enables endoscopists to predict polyp histology and make informed decisions for improved real-time settings.^[3–7]^

The previous classification of NBI International Colorectal Endoscopic (NICE), which relied on NBI with or without magnification, had limitations in differentiating between low and high-grade dysplasia and superficial and deep submucosal invasive lesions.^[1]^ This differentiation is clinically valuable for deciding treatment options, as the depth of invasion of malignant neoplasia should be evaluated using samples obtained en bloc by EMR or ESD.^[4,5,8]^ Magnified endoscopy with NBI has been reported to help differentiate between adenomas and non-neoplastic lesions by estimating the depth of cancer invasion.^[9,10]^ The Japan NBI Expert Team (JNET) recently proposed a classification system based on NBI with magnification.^[11]^ This allows a more detailed assessment of the degree of dysplasia and the extent of invasion in colorectal polyps. JNET classification helps endoscopists select appropriate treatment strategies, such as EMR for low-grade dysplasia (JNET 2A) and ESD for high-grade dysplasia and superficial submucosal invasive cancer (JNET 2B).^[4,12,13]^ Differentiation among low-grade dysplasia (LGD), high-grade dysplasia (HGD), and sSM by JNET classification may become essential because less invasive endoscopic treatment is becoming more widespread internationally. Although the effectiveness of JNET classification with optical magnification has been demonstrated in Japanese research, the availability of optical magnification systems outside Japan is limited, with alternative dual focus magnification being more commonly utilized.^[3,14,15]^ Dual-focus (DF) is a magnification mode that can provide images similar to optical zoom magnifying images by simply pushing a button.^[13,15]^ In Vietnam, the diagnostic value of the JNET classification with NBI-DF is still under investigation and the adoption of the classification outside Japan is limited. Therefore, this study aimed to evaluate the diagnostic precision of the JNET classification with NBI-DF in predicting the histology of colorectal polyps in a Vietnamese center.

## 2. Materials and methods

### 2.1. Study design and patients

A cross-sectional study was conducted among patients who underwent colonoscopy at the University Medical Center, Ho Chi Minh City, Vietnam, between October 2021 and March 2023. We enroll patients who met the following criteria: aged ≥ 18 years; observed by NBI-DF combined with JNET classification; and underwent en bloc resection with snare polypectomy, biopsy, EMR, ESD or surgical operation. The exclusion criteria were as follows: inadequate intestinal preparation or incomplete colonoscopy in patient preparation according to the Boston Bowel Preparation Scale (BBPS) with a total score of < 6 and each region score of < 2; withdrawal time less than 6 minutes; patients with polyposis syndromes, inflammatory bowel disease, or pregnancy; patients who were unwilling to participate in the research; polyps with unclear endoscopic images, those that could not be resected, or those not recovered for histological analysis, and colorectal tumors > 5 cm due to the difficulty of obtaining a precise NBI diagnosis in such cases.

### 2.2. Data assessment

Demographic data, including sex and age, were collected.

Colonoscopies used Olympus CFH190L colonoscopes and Evis-Exera III processors, with NBI-DF observation performed at a 1.4-fold electronic zoom (approximately 63-fold magnification).

The colonoscopy preparation involved patients who consumed 3000 mL of polyethylene glycol (PEG) (Fortrans^®^, Beaufour Ipsen Industrie, France) 5 hours before the procedure. Four experienced endoscopists (NQL, DML, VTTL, and TTTL) performed at least 3000 colonoscopic procedures in the last 5 years. They had an adenoma detection rate of more than 25%, proficiently employing dual focus mode, resulting in high-performance colonoscopies. During the endoscopic procedure, they evaluate the basic characteristics of the polyp on white light examination and give a real-time optical diagnosis with NBI-DF. Lesions were classified into JNET types 1, 2A, 2B, or three according to specific histological features, focusing on vessel and surface patterns (see Figure S1, Supplemental Digital Content, http://links.lww.com/MD/N82, which illustrates the JNET classification). If the lesion has many features of more than two types of JNET type, the highest was used to predict. All endoscopists received a lecture on JNET classification just before the study. An independent observer (TMH) recorded the polyp features and predictions. The polyps identified in white light were documented for location, macroscopic shape, and size relative to open biopsy forceps or snares.^[16]^ The locations of polyps were divided into two categories: distal (located in the descending colon, sigmoid colon, or rectum) and proximal (located in the ascending colon, transverse colon, and cecum). To determine the size of the lesions, the dimensions of the polyps were compared to those of the biopsy forceps (2.3 mm when closed, ENDO-FLEX) and the polypectomy snare (10 mm when open, SnareMaster®, Olympus).

### 2.3. Histopathological diagnosis

The tissue samples obtained from endoscopies were preserved in formalin for 24 hours, followed by standard processing procedures, including dehydration, paraffin embedding, sectioning, and hematoxylin-eosin (H/E) staining. Two experienced gastroenterology pathologists (HML and DTVN), blinded to endoscopic predictions, reviewed all resected lesions. Any disagreements were discussed to achieve consensus. Histological assessments were based on the World Health Organization and Vienna classifications.^[17,18]^ According to Japanese guidelines, carcinomas with vertical invasion up to 1000 µm in the submucosal layer were labeled sSM carcinoma. Compared, those with invasion ≥ 1000 µm were categorized as deep submucosal invasive (SM-d) carcinoma.^[19]^

### 2.4. Statistical analysis

Data were recorded in an MS Excel worksheet and analyzed using IBM SPSS 23.0 software. Categorical variables were presented as percentages, frequencies, and proportions, while continuous variables were expressed as means, and the median was used for non-normally distributed continuous variables. Sensitivity, specificity, Positive predictive values (PPV), negative predictive values (NPV), and accuracy of the JNET classification were assessed for each category. Kappa values measured the consistency between predicted pathology and actual pathology (poor, <0.20; fair, 0.21–0.40; moderate, 0.41–0.60; substantial, 0.61–0.80; excellent, 0.81–1.00). Statistical analysis was performed using Fisher’s exact test, with significance at *P* < .05.

Sample size determination

We are examining the specificity of JNET classification in diagnosing concordant pathology. We used the formula $\;n_{non-diesease}=\frac{Z_{1-\alpha /2}^{2}\;Spec\left(1-Spec\right)}{d^{2}}$ and $N=\frac{n_{non-diesease}}{1-Prevalence}$ where the desired precision d=0.05, confidence level 100(1 − α) = 95%. According to Sumimoto et al, the specificity of JNET-1, 2A, 2B, and 3 were 99.9%, 92.7%, 82.8%, and 99.8%, respectively, and the prevalence of hyperplastic polyp (HP)/SSP, LGD, HGD/superficial submucosal invasive (SM-s), SM-d were 4.6%, 65.6%, 22.4%, 7.2%.^[4]^ The required sample sizes for analysis were 17, 302, 282, and 4 cases. Thus, we need at least 302 cases for final analysis.

### 2.5. Endpoints

The primary endpoint was the relationship between the JNET type and the histopathological diagnosis of all polyps. The secondary endpoints were the diagnostic performance in differentiating neoplasia (type 2A) from nonneoplasia (type 1), malignant neoplasia (type 2B, 3) vs. benign neoplasia (type 2A), and invasive deep submucosal cancer (type 3) from other neoplasia (type 2A, 2B).

### 2.6. Ethics

This study was approved by the Ethnic Board of University Medical Center Ho Chi Minh City, Viet Nam (62/GCN-HĐĐĐ). The study protocol conforms to the ethical guidelines of the 1975 Declaration of Helsinki, as reflected in prior approval by the institution’s human research committee.

## 3. Results

### 3.1. Characteristics of the enrolled patient and colorectal lesions

**Figure 1.**
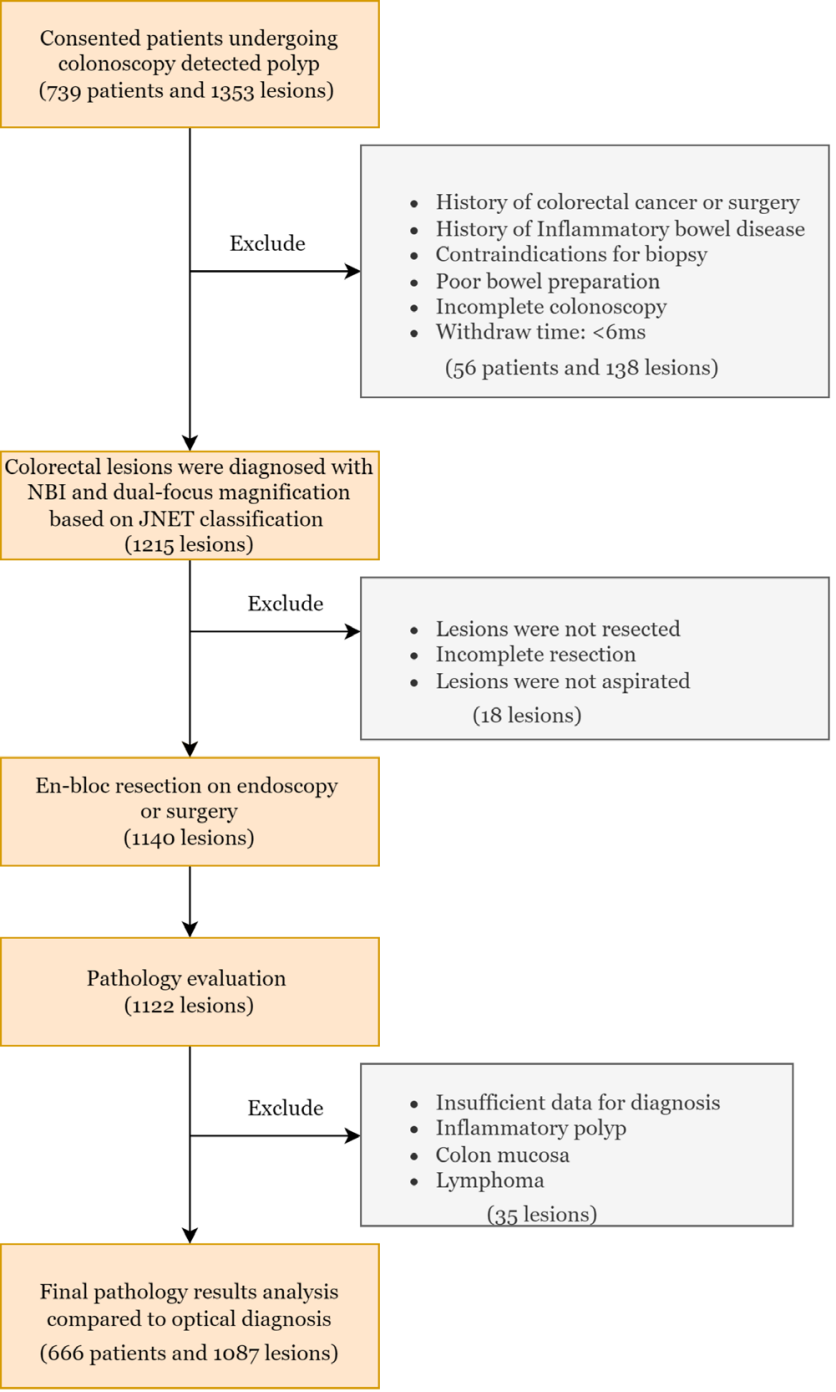
Flow diagram of the study

**Figure 2.**
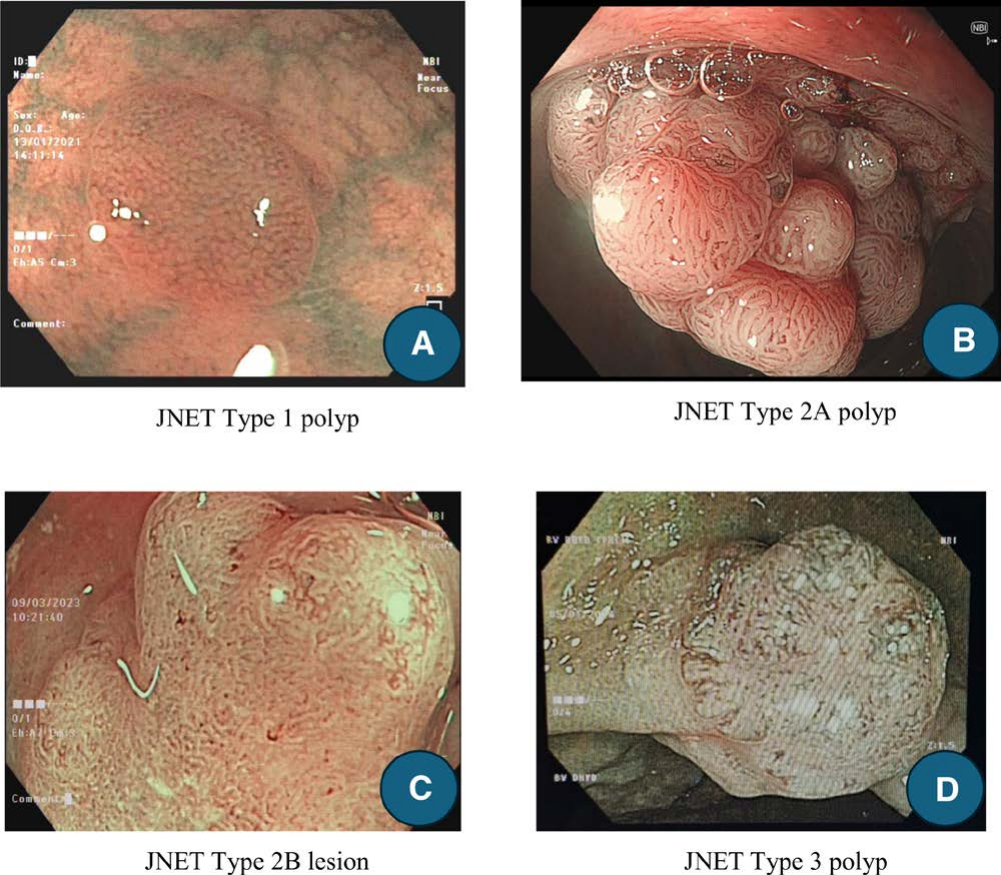
Characteristics of all colorectal polyps. (A) JNET type 1, (B) JNET Type 2A, (C) JNET Type 2B, and (D) JNET Type 3 polyps.

There were 739 patients with 1353 lesions enrolled, and 1087 lesions were included in the final analysis (Fig. 1). The mean age of patients was 59 ± 1.11, and the male/female ratio was 1.44/1. The clinicopathological characteristics of the lesions are shown in Table 1. The median size of the lesion was 8.6 mm (1–40 mm), with non-polypoid lesions being the most frequently observed morphology (374 lesions, 34.3%). The sigmoid colon was the most common location, with 395 lesions (52%). After NBI analysis, the lesions were classified according to the JNET classification: 290, type I; 70,7 type 2A; 75 type 2B; and 15 type 3 (Fig. 2). The most prevalent histological findings were LGD in 695 lesions (63.9%), while advanced histology (high-grade dysplasia to submucosal invasion) was observed in 96 lesions (8.8%).

**Table 1:** Clinicopathological characteristics of all lesions.

Characteristic		N	%
No. of lesions		1067	100
Location	Rectum	197	18.1
	Sigmoid colon	395	36.3
	Descending colon	122	11.2
	Transverse colon	186	17.1
	Ascending colon	143	13.2
	Ceacum	44	4.1
Size group	<10 mm	636	58.5
	≥10 mm	451	41.5
Lesion in size (mm), median (range)		8.6 (1–40)	
Morphology* (n, %)	0-Is, Is + IIc	541	49.8
	0-Ip	171	15.8
	0-IIa, IIa + IIc	374	34.3
	0-IIb	001	00.1
Treatment procedure (n, %)	Cold forceps polypectomy	455	41.7
	Snare polypectomy	419	38.5
	Cold snare	69	06.3
	pEMR	67	6.1
	En bloc EMR or ESD	62	5.6
	Surgical resection	15	01.8
Histology findings (n, %)	Hyperplastic	274	25.2
	Sessile serrated lesion	21	01.9
	Tradition serrated adenoma	9	02.7
	Tubular adenoma	626	57.6
	Villotubular adenoma	005	00.5
	Villous adenoma	122	11.2
	Carcinoma	029	00.9
Dysplasia (n, %)	Negative for neoplasia	296	27.2
	Low grade intramucosal neoplasia	695	63.9
	High grade intramucosal neoplasia	66	6.2
	Shallow submucosal invasive cancer (<1000 µm)	09	0.8
	Deep submucosal invasive cancer (≥1000 µm)	21	1.9

EMR *=* endoscopic mucosal resection, ESD *=* endoscopic submucosal dissection, pEMR *=* piecemeal endoscopic mucosal resection.

* According to the Paris classification.

### 3.2. Diagnostic value of NBI-DF combined with JNET classification

The correlation between JNET classification and final histology is described in Table 2. The overall concordance with the JNET classification was 86.9%. Across all types of JNET, the precision in the prediction of histology was lowest in the JNET-2B group (54.6%, 41 of 75 cases), demonstrating a low κ coefficient of agreement of 0.51. On the contrary, the highest precision was observed in JNET-3 (93.3%, 14 of 15 cases), reflecting a substantial κ coefficient of agreement of 0.78. When stratified by size, lesions less than 10mm were not diagnosed as JNET type 3. For polyps smaller than 10mm and larger than 10mm, the highest concordance was observed in JNET type 1 (87.8–100%), while the lowest was in JNET type 2B (0–54%). The diagnostic values for the JNET classification are presented in Table 3. Regarding sensitivity, JNET types 1, 2A, 2B, and 3 exhibited rates of 86.5%, 91.9%, 54.7%, and 100%, respectively. Regarding specificity, the values for JNET type 1, 2A, 2B, and 3 lesions were 95.7%, 81.4%, 8%, and 99.1%, respectively. PPV were 91%, 90.7%, 50%, and 90% for Types 1, 2A, 2B, and 3, respectively, while NPV were 100%, 85.7%, 92.6%, and 96%, respectively. The overall diagnostic precision was 98.5%, 88.4%, 83%, and 93.5% for JENT type 1, 2A, 2B, and 3, respectively. When stratified by size, JNET-1 demonstrated the highest PPV in the group with a size greater than 10 mm, while JNET-2A achieved the highest PPV in the group with less than 10 mm.

**Table 2:** Relationship between JNET classification and histopathological diagnosis in real-time endoscopy.

JNET classification	Size	n	Histological findings					Cohen’s kappa
			HP, SSL	LGD	HGD	Carcinoma		
						SM-s	SM-d	
Type 1	All	290	256	34	0	0	0	0.83
	≥10 mm	12	12	0	0	0	0	0.51
	<10 mm	278	244	34	0	0	0	0.83
Type 2A	All	707	40	634	32	1	0	0.73
	≥10 mm	351	21	305	25	0	0	0.57
	<10 mm	356	19	319	7	1	0	0.82
Type 2B	All	75	0	27	34	7	7	0.51
	≥10 mm	73	0	25	34	7	7	0.52
	<10 mm	2	0	2	0	0	0	–
Type 3	All	15	0	0	0	1	14	0.78
	≥10 mm	15	0	0	0	1	14	0.77
	<10 mm	0	0	0	0	0	0	–
Total		1087	296	695	66	9	21	

HP = hyperplastic polyp, HGD = high-grade dysplasia, LGD = low-grade dysplasia, SSL = sessile serrated lesion, SM-d = submucosal invasive carcinoma of deeper than 1000 μm, SM-s = submucosal invasive carcinoma of less than 1000 μm.

**Table 3:** Diagnostic values for each JNET type stratified by polyp size.

JNET type	Size	Sens (%) CI-95%	Spec (%) CI-95%	PPV (%) CI-95%	NPV (%) CI-95%	Accuracy (%) CI-95%
JNET-1	All	86.5 82.1–90.2	95.7 94.1–97.0	88.3 84.4–91.3	95.0 93.4–96.2	93.2 91.5–94.6
	≥10 mm	36.4 20.4–54.9	100 99.2–100	100 73.5–100	95.4 94.2–06.4	95.6 93.3–97.2
	<10 mm	92.8 89.0–95.6	90.9 87.5–93.6	84.8 83.9–90.8	94.7 92.1–96.5	91.7 89.2–93.7
JNET-2A	All	91.9 88.9–93.2	81.4 77.2–85.1	90 87.6–91.5	84.0 80.4–87.0	87.7 85.6–89.6
	≥10 mm	92.2 88.7–94.8	61.7 52.4–70.4	86.9 84.1–89.3	74.0 65.7–80.7	84.1 80.3–87.3
	<10 mm	89.9 86.2–92.8	90.4 86.3–93.6	92.2 89.2–94.4	87.6 83.8–90.6	90.1 87.5–92.3
JNET-2B	All	54.7 42.8–66.2	96.6 95.3–97.7	54.7 45.0–64.1	96.6 95.7–97.4	93.7 92.2–95.1
	≥10 mm	61.2 48.5–72.7	91.7 88.5–94.2	56.2 46.6–65.3	93.1 90.9–94.8	87.1 83.7–90.1
	<10 mm	–	96.7 98.7–99.9	–	100 99.4–100	–
JNET-3	All	100 76.8–100	99.9 99.5–100	93.3 65.9–99.0	100 99.7–100	99.9 99.5–100
	≥10 mm	100 76.84–100	99.8 98.7–99.9	93.3 66.4–99.0	100 99.2–100	99.8 98.8–99.9
	<10 mm	–	–	–	–	–

CI = confidence interval, NPV = negative predictive value, PPV = positive predictive value, Sen = sensitivity, Spec = specificity.

### 3.3. JNET diagnostic performance and treatment strategy guidance

When differentiating neoplasia (JNET-2A) from nonneoplasia (JNET-1), sensitivity and NPV were 97.8% and 88.3%, respectively. Diagnostic performance was markedly higher in lesions larger than 10 mm compared to those smaller than 10 mm (93.8% vs 92.3% and 100% vs 88.3%, respectively).

Diagnostic accuracy, specificity and NPV to distinguish malignant neoplasia (types 2B and 3) from benign neoplasia (type 2A) were 92.3%, 95.9%, and 95.1%, respectively. However, the sensitivity was lower at 64.1%. For deep SM-d carcinoma from other neoplasms, specificity and PPV were remarkably high at 99.8% and 93.3%, respectively (Table 4).

**Table 4:** Diagnostic performance of the JNET classification for the treatment strategy.

Determination of diagnosis	Size group	Sens (%) CI-95%	Spec (%) CI-95%	PPV (%) CI-95%	NPV (%) CI-95%	Accuracy (%) CI-95%
Neoplasia (type 2A) vs non neoplasia (type 1)	All	94.9 92.9–96.5	86.5 82.1–90.2	94.1 92.3–05.5	88.3 84.4–91.3	92.3 90.5–93.9
	≥10 mm	100 98.8–100	36.4 20.4–54.9	93.6 86.6–93.2	100 73.5–100	93.8 90.7–96.11
	<10 mm	90.4 86.8–93.2	93.2 89.7–95.9	94.4 91.6–96.3	88.5 84.8–91.4	91.7 89.2–93.7
Malignant neoplasia (type 2B, 3) vs benign neoplasia (type 2A)	All	64.1 53.5–73.8	95.9 94.1–97.3	68.6 59.4–76.5	95.1 93.6–96.2	92.1 89.9–93.9
	≥10 mm	65.8 53.7–76.5	92.4 89.1–95.1	65.8 56.0–74.3	92.4 89.7–94.4	87.6 84.0–90.7
	<10 mm	0 0–36.9	99.4 97.8–99.2	0	97.6 97.5–97.6	96.9 94.5–98.5
Deep submucosal invasive cancer (type 3) from other neoplasia (type 2A, 2B)	All	66.7 43.1–85.4	99.8 99.2–100	93.3 65.9–99.0	99.1 98.2–99.5	98.9 97.8–99.5
	≥10 mm	66.7 43.1–85.4	99.8 98.4–100	93.3 65.9–99.0	98.0 96.4–98.9	97.8 95.7–99.1
	<10 mm	–	100 98.9–100	–	100 98.9–100	–

CI = confidence interval, NPV = negative predictive value, PPV = positive predictive value, Sen = sensitivity, Spec = specificity.

### 3.4. Characteristics of misclassified lesions and sessile serrated lesions

In total, 61 neoplastic lesions were evaluated as JNET type 1, with a median size of 3.6 mm (range, 1–10 mm). Approximately two-thirds of these lesions were located in the proximal region and exhibited macroscopic characteristics of type 0–IIa + IIc. Furthermore, 27 LGN lesions were classified as JNET type 2B, with approximately two-thirds found in the distal colon, showing macroscopic features 0 to Is, Is + IIc, and a median size of 27 (2–15 mm). In addition, 33 Tis (HGD) lesions extending to the submucosa were classified as JNET type 2A, most located in the distal colon, with a median size of 21.1 mm (range 12–40 mm). Regarding the JNET-2B group, 7 out of 74 cases (9.3%) showed evidence of deep invasive cancer. No cases without neoplasia were predicted as JNET-3 (Table 5). When comparing the characteristics between nondysplastic and dysplastic serrated lesions, dysplastic lesions were markedly larger (12 ± 6.8 vs 4 ± 1.7mm, *P* = .002). They exhibited more JNET-2A-type characteristics compared to non-dysplastic lesions (12 vs 3, *P* < .001) (see Table S1, Supplemental Digital Content, http://links.lww.com/MD/N82, which illustrates endoscopic features of the sessile serrated lesions).

**Table 5:** Representative lesions misclassified by JNET classification.

JNET type		Histology findings					
		Neoplastic	Non-neoplastic	LGD	HGD/SM-s	HGD/SM-s	SM-d
		Type 1	Type 2A	Type 2B	Type 2A	Type 3	Type 2B
No of lesion		61	30	27	33	1	7
Size, median (range)		3.6 (1–10)	8.0 (2–15)	22.1(8–40)	21.1(12–40)	40	17.9 (15–25)
Location	Proximal	41	16	18	8	0	1
	Distal	20	14	9	25	1	6
Macroscopic type	Ip	0	5	15	10	1	2
	Is, Is + IIc	20	19	12	23	0	5
	IIa, IIa + IIc	41	6	0	0	0	0

HP = hyperplastic polyp, HGD = high-grade dysplasia, LGD = low-grade dysplasia, SSL = sessile serrated lesion, SM-d = submucosal invasive carcinoma of deeper than 1000 μm, SM-s = submucosal invasive carcinoma of less than 1000 μm.

## 4. Discussion

To our knowledge, this is the first study to validate the diagnostic performance of the JNET classification with NBI-DF in a Vietnamese real-time setting. Our study demonstrated that DF magnification helps Vietnamese endoscopists achieve high diagnostic performance in the optical diagnosis of Type 1, Type 2A, and Type 3 lesions. However, the correlation was poor for type 2B, suggesting that additional tests or imaging techniques may be required. Furthermore, this classification with NBI-DF helps rule out early non-neoplastic lesions and accurately diagnose SM-d cancer, potentially avoiding unnecessary polypectomies and surgeries.

**Figure 3.**
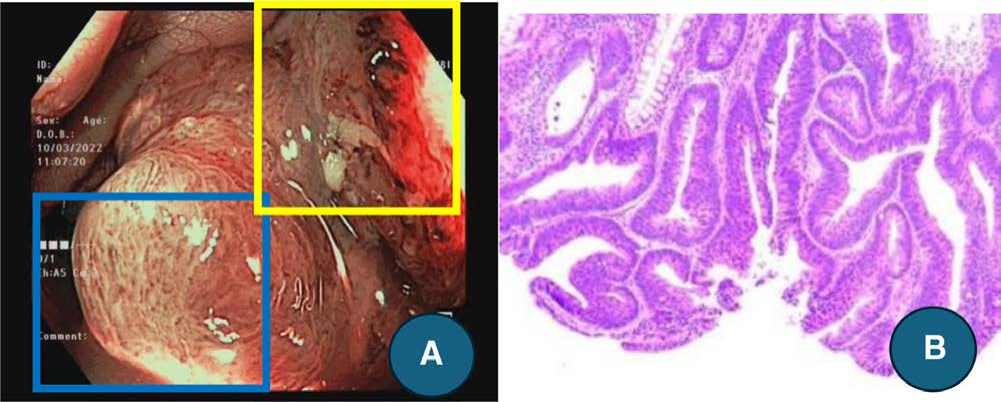
Illustration of mixed-type JNET. A lesion (0-Is + IIc) with two distinct areas represented by JNET-2A (blue box) and JNET-2B (yellow box) presented a challenge for endoscopists during the evaluation of the lesion (A). The final histology revealed a high-grade villotubular adenoma (B).

Our results for JNET-1 lesions showed very high specificity (95.7%) and PPV (95%). Particularly with lesions larger than 10 mm, specificity and PPV could reach 100%. These figures suggest that a lesion diagnosed as non-neoplastic can be classified with high precision and reliability, and resection should be considered.^[3,4,8,13,20]^ Regarding LGD lesions, JNET-2A showed a lower specificity (81.4%) than other JNET groups, but had the highest sensitivity (91.9%), particularly with sizes > 10 mm. Therefore, the JNET classification helps to detect LGD early. Importantly, differentiating JNET-2A lesions from JNET-1 is vital to determine the appropriate endoscopic treatment.^[20]^ Our results suggest that the JNET classification was highly discriminatory, with a sensitivity of 97.8% and a precision of 94.3% in differentiating between JNET-2A and JNET-1. Type 2A lesions are often considered suitable for endoscopic resection, while Type 1 lesions can be left without discarding, especially those at a size ≤ 5 mm.^[3,4,8,13]^ However, in our study, 33 lesions with a histological classification of HGN to SM-s were misclassified as JNET Type 2A. These are frequently protruding lesions (0-Ip, 0-Is, Is + IIc) of greater size in the distal colon. These lesions could potentially be advanced malignancies disguised as adenomas, requiring thorough endoscopic investigation (Fig. 3).^[13]^ There is concern about SSL (sessile serrated lesion) with dysplasia (≤5 mm) as they can be classified as JNET-1 and left in situ. However, our study suggests that these lesions might be removed since they are usually classified as JNET-2A and have a significantly larger size (*P* < .05).

Regarding identifying high-grade dysplasia and superficial invasive cancer, JNET-2B exhibited the lowest sensitivity (54.7%) compared to other JNET groups. The concordance rate of JNET-2B for neoplastic histology was also only 54.6%. These results with dual focus magnification are similar to those of Sumimoto (61.9%) and Koyama (39.1%), who used traditional zoom optical magnification.^[13,21]^ The correlation between JNET type 2B histopathology is not enough. In addition to hat, the small number of cases could influence the low PPV of JNET-2B. However, the accuracy, specificity, and NPV for differentiating high-risk lesions (JNET-2B and JNET-3) from low-risk lesions (JNET-2A) are 92.4%, 95.9%, and 95.1%, respectively. Consequently, the JNET classification with NBI-DF effectively helps identify high-risk lesions for the selection of en bloc resection (ESD, EMR, or surgery), but for a precise submucosal invasive diagnosis, we need further assessment.^[5,8,20]^

JNET-3 lesions exhibit high specificity and a PPV of more than 90%, as in other studies in Japan.^[5,8,20]^ JNET-3 is closely associated with deep invasive lesions. Therefore, these lesions are closely related to surgical requirements, and chromoendoscopy may not be necessary to assess the depth of invasion of the JNET-3 lesions. Furthermore, lesions without JNET Types 2B and 3 can rule out submucosal invasive carcinomas with high reliability.

The disparity between optical diagnosis and pathology can be attributed to various factors. First, complex lesions, often characterized by their large size, can harbor multiple pathological features, including HGD, SM-s carcinoma, and SM-d carcinoma. This complexity can lead to misidentification, as surface structures may exhibit HGD or SM-s carcinoma characteristics. At the same time, only a focal or deep part of the lesions can reveal features of SM-d carcinoma. Second, observation limitations due to the lesions’ large size or difficult-approached location can hinder endoscopists from thoroughly assessing the extent of the lesions, resulting in low sensitivity to diagnosis. Third, spontaneous or contact bleeding in some large lesions may lead to the adhesion of blood to the surface, influencing the judgment of pathological types. Therefore, obtaining a comprehensive view of lesions with a large size, particular locations, and pediculated shape is crucial for endoscopists to enhance accuracy in diagnosis. Additionally, for the diagnosis of depth invasion, endoscopists can combine crystal violet staining to assess vessel and surface patterns.^[3,13,21]^ This is due to a previous study demonstrating that crystal violet staining for the pit pattern is a more accurate predictor than magnified NBI for determining cancer depth.^[11,22]^ If additional information on the pattern of the pits using crystal violet is inaccessible, the decision-making process could consider evaluating a combination of macroscopic types based on the Paris classification and the morphology of the patterns of combined classifications like CONECCT.^[23]^

In our study, we cannot compare the agreement between the observers between our endoscopists during the real-time evaluation. However, several studies have demonstrated the reliability and consistency of the JNET classification. An Indian study by Ahire et al found substantial agreement (κ = 0.76) in classification, with 3.5% disagreements in 144 polyps, mainly type 2B.^[24]^ Another investigation of 246 colorectal lesions showed κ = 0.72 agreement between observers among three experienced endoscopists, with disagreements in only 1% of cases.^[22]^ Furthermore, a Japanese study observed moderate interobserver agreement among three observers, with vessel/surface pattern κ values of 0.52/0.52 and a complete concordance rate of 67%.^[8]^

Many studies have highlighted the efficacy of DF in diagnosing colorectal tumors. In a review of 100 cases of colorectal polyps, NBI-DF proved to be more effective in distinguishing neoplastic from non-neoplastic lesions than white-light endoscopy or non-magnifying NBI. Another study evaluating 149 colorectal polyps, including 38 lesions larger than 10 mm, found that NBI-DF, based on modified Sano classification, achieved a diagnostic precision of 96.6%. Furthermore, endoscopic predictions for rectosigmoid diminutive polyps demonstrated a 100% negative predictive value for adenomatous histology.^[14]^ However, these studies only use the NICE classification. Recently, in Japan, Koyama analyzed 557 images of lesions, highlighting the superiority of NBI-DF, with results supporting its high diagnostic accuracy in predicting the histology of HP/SSL, LGD, HGD, and SM-s, with specificities of 98.6%, 76.5%, 99.1%, and 99.5%, respectively.^[3]^

Based on these large-scale data from 1087 lesions in a clinical setting, our study underscores the high diagnostic performance of the JNET classification in a Vietnamese setting. This research provides pioneering real-time endoscopy data using the JNET classification from a Vietnamese center. The prospective nature of our study, coupled with a thorough polyp characterization by a highly experienced endoscopist and histopathological evaluation by two gastrointestinal pathology specialists, supports the credibility of our findings. These results show that Vietnamese endoscopists can effectively employ this classification in real-time. Our result was consistent with other validation studies conducted in Japan.^[3,4,7,8,25,26]^ Although previous studies have evaluated the diagnostic precision of JNET, most were retrospective and relied on previous still images with traditional optical zoom in Japan, our study investigated the efficacy of dual focus magnification in Vietnamese colonoscopy centers (Table 6).^[4,7,8,20]^ The disparity may arise from differences in the population, endoscopic systems, endoscopist experience, and the evaluation process.

**Table 6:** Diagnostic performance of the Japan narrow band imaging expert team classification in NBI.

Author	Year	Country	No. lesion	Study design	Real-time	Magnification mode	JNET 1		JNET2A		JNET2B		JNET3	
							Acc (%)	PPV (%)	Acc (%)	PPV (%)	Acc (%)	PPV (%)	Acc (%)	PPV (%)
Sumimoto^[21]^	2017	Japan	2933	Retrospective	X	Optical zoom	99.3	97.5	77.1	98.3	78.1	50.9	96.6	95.2
Komeda^[8]^	2017	Japan	199	Retrospective	No	Optical zoom	98.5	92.3	90.9	90.3	87.4	67.3	94.0	100
Kobayashi^[13]^	2019	Japan	1402	Retrospective	No	Optical zoom	93	96	87	92	93	26	98	93
Hirata^[20]^	2019	Japan	6138	Retrospective	No	Optical zoom	89.3	87.9	85.8	86.6	96.6	44.7	99.6	94.7
Ahire^[24]^	2020	Indian	90	Prospective	X	Non-manification	96	90	82	90	90	38	97	78
Mareedu^[27^^]^	2022	Indian	158	Prospective	No	Dual focus	98.5	91	88.5	90.7	83	50	93.5	90
Koyama^[3]^	2022	Japan	557	Retrospective	No	Dual focus	95.3	87.3	91.9	80.6	95.5	45.0	98.9	50.0
Saito^[23]^	2023	Japan and Europe	150	Retrospective	No	Optical zoom	93–93.5	NA	62.1–65.1	NA	55.1–59.5	NA	86.9–88.4	NA
Our study	2023	Vietnam	1087	Prospective	X	Dual focus	93.2	88.3	87.7	90	93.7	54.7	99.9	93.3

Acc = accuracy, NA = not available, PPV = positive predictive value.

Even with the significant insights gained from our study, certain limitations warrant consideration. The research was carried out in a single tertiary center, which could limit the applicability of our findings to a broader context. To foster the broad clinical adoption of JNET classification, more studies are essential involving less experienced physicians and those in community settings in Vietnam. However, a recent study found that trainees with no previous colonoscopy experience showed comparable diagnostic accuracy to experts after receiving a single 20-minute lecture on the JNET classification.^[7]^ Second, the endoscopies were performed by a single endoscopist, which could lead to interindividual variations in interpreting the NBI-DF findings. Although interobserver bias was unavoidable, it was minimized as all endoscopists participating in this study had experience, and a local training workshop was held before recruiting patients. Third, potential concerns may arise due to the limited sample size for high-grade dysplasia, in situ carcinoma, and invasive carcinoma polyps. These concerns might affect the reliability of the PPV and NPV of JNET-2B and JNET-3. Finally, the chromoendoscopy was not performed further in our research, especially for lesion JNET-2b, due to a lack of resources. We will further conduct a combination of virtual and real chromoendoscopy to thoroughly evaluate the clinical utility of JNET classification in our setting.

In conclusion, JNET classification using DF magnification performed well in predicting colorectal polyp histology. JNET may contribute to appropriate treatment choices and avoid unnecessary surgeries. However, further research is needed in the Vietnamese setting due to the lower specificity and PPV of type 2B.

## Acknowledgments

The authors thank the staff at the Gastrointestinal Endoscopy Department for their support in this study. Additionally, we are grateful to Dr Thinh Ong from the Mathematical Modelling group at the Oxford University Clinical Research Unit in Ho Chi Minh City, Vietnam, for providing valuable statistical consultation.

## Author contributions

**Conceptualization:** Nhan Quang Le, Nghia Quang Le.

**Data curation:** Nhan Quang Le, Tien Manh Huynh, Diem Thi Ngoc Vo, Huy Minh Le, Vy Thao Ly Tran, Truc Thanh Le Tran, Luan Minh Dang, Nghia Quang Le.

**Formal analysis:** Nhan Quang Le, Tien Manh Huynh, Nghia Quang Le.

**Funding acquisition:** Nhan Quang Le, Nghia Quang Le.

**Investigation:** Nhan Quang Le, Diem Thi Ngoc Vo, Huy Minh Le, Luan Minh Dang.

**Methodology:** Nhan Quang Le.

**Project administration:** Nhan Quang Le.

**Resources:** Nhan Quang Le, Tien Manh Huynh.

**Supervision:** Luan Minh Dang, Nghia Quang Le.

**Validation:** Tien Manh Huynh, Vy Thao Ly Tran.

**Visualization:** Tien Manh Huynh, Truc Thanh Le Tran.

**Writing – original draft:** Nhan Quang Le, Tien Manh Huynh, Nghia Quang Le.

**Writing – review & editing:** Nhan Quang Le, Tien Manh Huynh, Nghia Quang Le.

## Supplementary Material
