# Supplementary material for: Diagnostic performance of the Japanese Narrow-band imaging expert team classification system using dual focus magnification in real-time Vietnamese setting
Source: Medicine (Baltimore). 2024 Jul 5;103(27):e38752. doi: 10.1097/MD.0000000000038752 (PMC11224830; doi:10.1097/MD.0000000000038752)
Supplement: Supplementary file 1 [file medi-103-e38752-s001.docx]

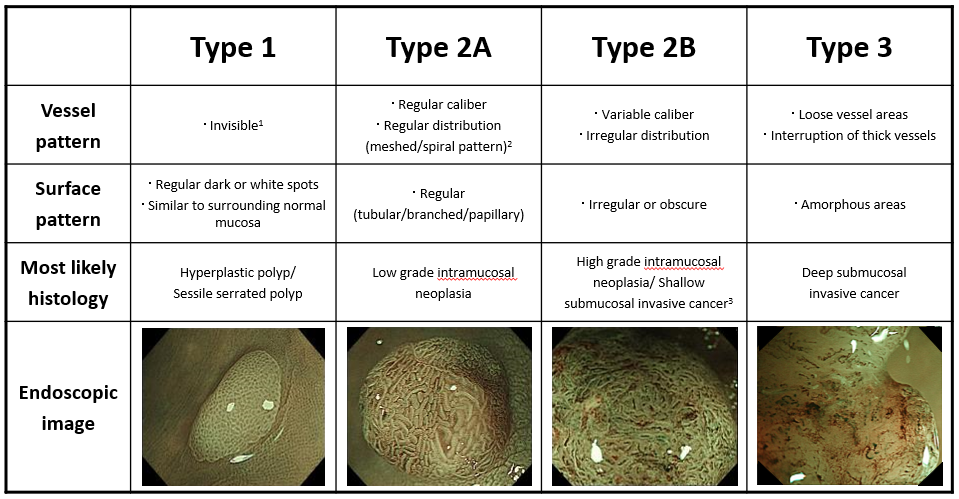


**Supplemental figure 1. The Japan Narrow Band Imaging Expert Team classification**

^1^If visible, the caliber in the lesion is similar to surrounding normal mucosa.

^2^Micro-vessels are often distributed in a punctate pattern and well-ordered reticular or spiral vessels may not be observed in depressed lesions. ^3^Deep submucosal invasive cancer may be included.

**Supplemental Table 1.** **Endoscopic features of the sessile serrated lesions**

| **Characteristic** | | **No dysplasia**  **(n=9)** | **Low-grade dysplasia**  **(n=12)** | **p*** |
| --- | --- | --- | --- | --- |
| **Location** | **Proximal** | 2 | 5 | .64 |
|  | **Distal** | 7 | 7 |  |
| **Size of tumor** | | 4 ±1.7 | 12 ± 6.8 | .002 |
| **Macroscopic shape** | **0-Is, 0-Is+0-IIc** | 4 | 9 | .07 |
|  | **0-Ip** | 0 | 3 |  |
|  | **0-IIa** | 5 | 0 |  |
| **Mucus cap** | | 3 | 5 | 1 |
| **JNET classification** | **JNET-1** | 6 | 0 | .001 |
|  | **JNET-2A** | 3 | 12 |  |

(*)Fisher extract test
